# Data of the natural and pharmaceutical angiotensin-converting enzyme inhibitor isoleucine-tryptophan as a potent blocker of matrix metalloproteinase-2 expression in rat aorta

**DOI:** 10.1016/j.dib.2016.06.059

**Published:** 2016-07-05

**Authors:** Irakli Kopaliani, Melanie Martin, Birgit Zatschler, Bianca Müller, Andreas Deussen

**Affiliations:** Department of Physiology, Faculty of Medicine, Technische Universität Dresden, Fetscherstraße 74, Dresden, Germany

**Keywords:** Isoleucine-Tryptophan, Angiotensin-converting enzyme, Angiotensin II, Matrix metalloproteinase

## Abstract

The present data are related to the research article entitled “Whey peptide isoleucine–tryptophan inhibits expression and activity of matrix metalloproteinase-2 in rat aorta” [1]. Here we present data on removal of endothelium from aorta, endothelium dependent aortic relaxation and inhibition of expression of pro-MMP2 by di-peptide isoleucine–tryptophan (IW). Experiments were performed in rat aortic endothelial cells (EC) and smooth muscle cells (SMC) *in vitro*, along with isolated rat aorta *ex vivo*. The cells and isolated aorta were stimulated with angiotensin II (ANGII) or angiotensin I (ANGI). ACE activity was inhibited by treatment with either IW or captopril (CA). Losartan was used as a blocker of angiotensin type-1 receptor. IW inhibited MMP2 protein expression induced with ANGI in a dose-dependent manner. IW was effective both in ECs and SMCs, as well as in isolated aorta. Similarly, captopril (CA) inhibited ANGI-induced MMP2 protein expression in both *in vitro* and *ex vivo*. Neither IW nor CA inhibited ANGII-induced MMP2 protein expression in contrast to losartan. The data also displays that removal of endothelium in isolated rat aorta abolished the endothelium-dependent relaxation induced with acetylcholine. However, SMC-dependent relaxation induced with sodium nitroprusside remained intact. Finally, the data provides histological evidence of selective removal of endothelial cells from aorta.

## **Specifications Table**

**Table:** 

Subject area	*Biology*
More specific subject area	*Bioactive natural peptides*
Type of data	*Image (histology, microscopy), graph, figure*
How data was acquired	*Microscope, gel electrophoresis, Mulvany Myograph (AD Instruments 610).*
Data format	*Analyzed*
Experimental factors	*Isolated rat aortic ECs and SMCs were stimulated with ANGII or ANGI. Isolated rat aorta was also stimulated similarly. Stimulations were performed with or without pretreatment with IW, CA or losartan. CA and losartan were used as positive controls for inhibition of ACE activity. In one set of experiments, endothelium was removed from isolated aorta in order to study specific effect of IW in SMCs. Efficient removal of endothelium was tested.*
Experimental features	*Western blot was used to quantitatively assess MMP2 protein expression. Hematoxylin–eosin staining was used for structural evaluation of the removal of endothelium from isolated aorta. Efficient removal of endothelium was functionally tested in Mulvany Myograph by assessing endothelium-dependent and independent relaxations.*
Data source location	Technische Universität Dresden, Germany
Data accessibility	Data are presented in this article

## Value of the data

• The data provides evidence of effectiveness of IW in prevention of adverse vessel remodeling.
• The data demonstrates that natural ACE inhibitory peptides may be similarly potent as pharmaceutical ACE inhibitors.
• The data provides a solid basis for further studies on the effects of tryptophan containing dipeptides on ACE mediated activation of the renin–angiotensin-system.

## 1 Data

**Fig. 1:**
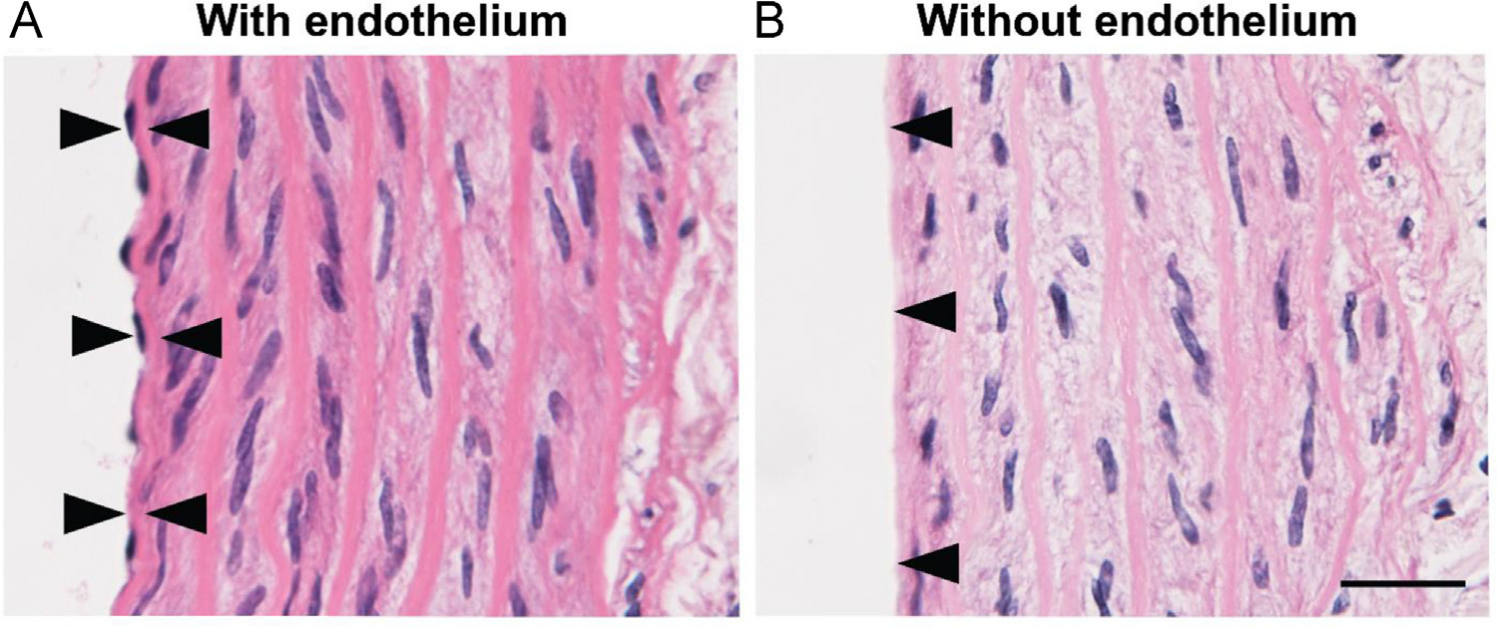
Representative images of aorta with (A) and without endothelium (B). In image A, opposite arrows point to the borders of the endothelial cell layer. In image B, arrows point to the intimal vessel surface where endothelial cells are missing. Paraffin sections of aorta were stained with hematoxylin–eosin as previously described [1]. Endothelium was removed with a cotton wire as described in methods. Scale bar=50 µm.

**Fig. 2:**
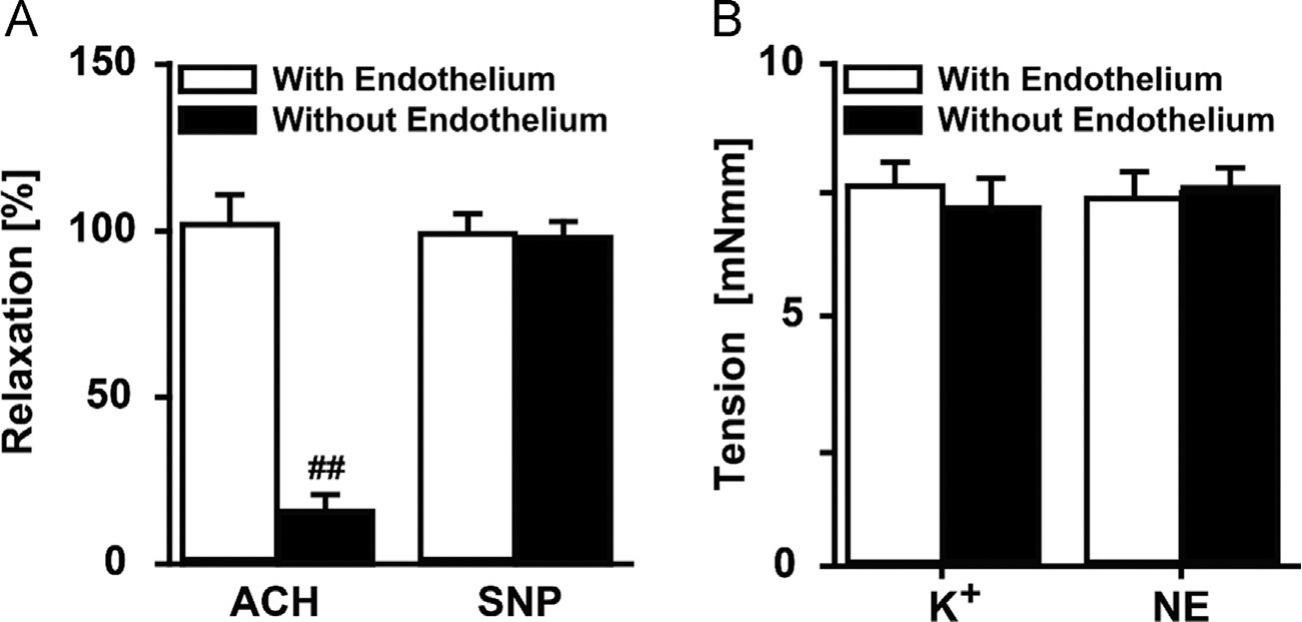
Functional validation of successful endothelial removal from aorta. (A) Removal of endothelium resulted in a nearly abolished endothelium-dependent relaxation tested with ACH, whereas endothelium-independent relaxation by SNP was fully maintained. (B) Removal of endothelium did not result in any impairment of development of tension in SMCs tested with either K^+^ or NE. ^##^*P<*0.05 *vs*. aorta with endothelium. Data is shown as mean±SE. *n*=5. Abbreviations: ACH – acetylcholine, SNP – sodium nitroprusside, NE – norepinephrine.

**Fig. 3:**
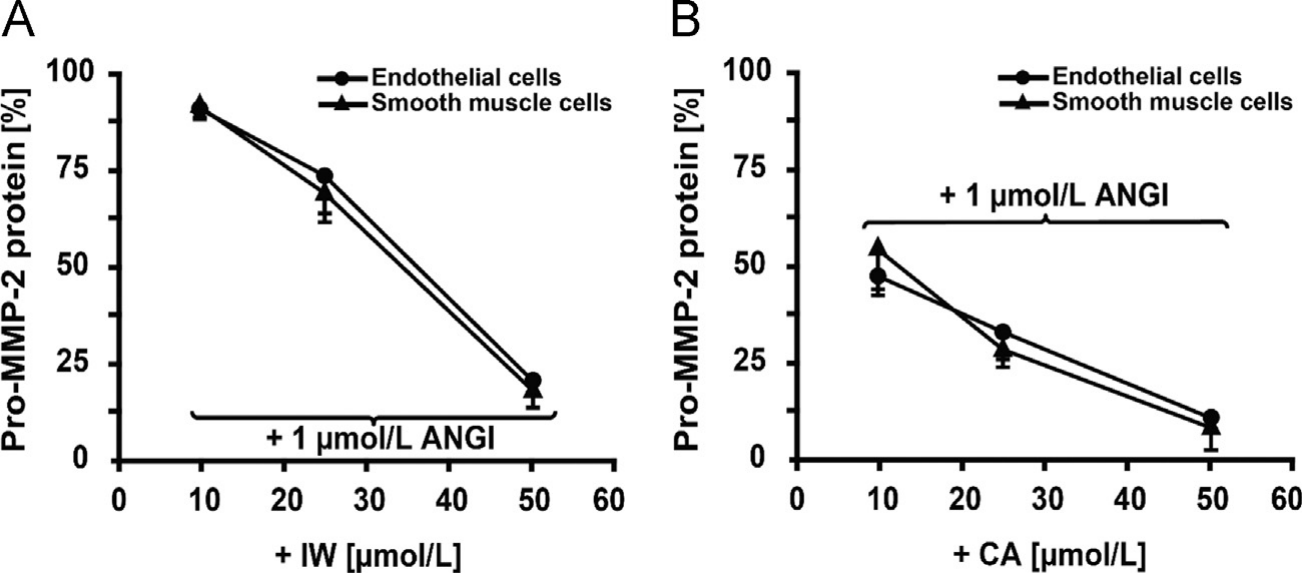
Dose–response curves for IW (A) and captopril (B) on pro-MMP-2 expression following stimulation with ANGI (8 h). 10, 25 and 50 µmol/L of IW or CA was used. Treatment was performed 30 min prior to ANGI stimulation (1 µmol/L). (A) While IW reduced the stimulatory effect of ANGI by 50% at approximately 35 µmol/L, (B) CA caused a similar inhibitory effect even at the lowest concentration tested (10 µmol/L). Data is shown as mean±SE. *n*=5. Abbreviations: IW – isoleucine–tryptophan, CA – captopril.

**Fig. 4:**
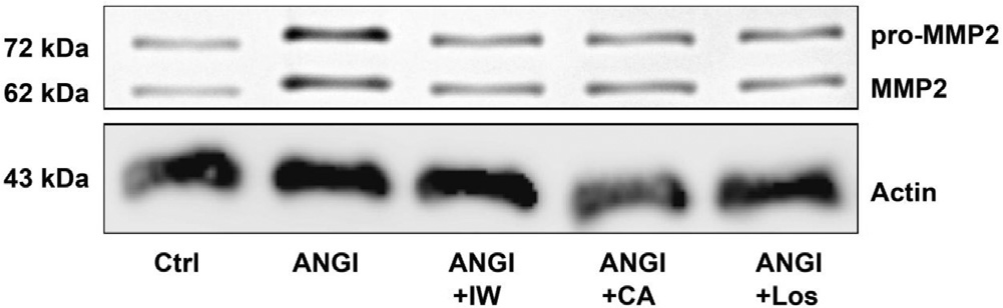
Representative western blot with latent (72 kDa) and active (62 kDa) forms of MMP2 in isolated rat aorta. Compared to untreated Ctrl, stimulation with ANGI resulted in an increase of both latent and active forms of MMP2. This effect was inhibited with IW, CA and Los. Abbreviations: Ctrl – untreated control, IW – isoleucine–tryptophan, CA – captopril, Los – losartan.

**Fig. 5:**
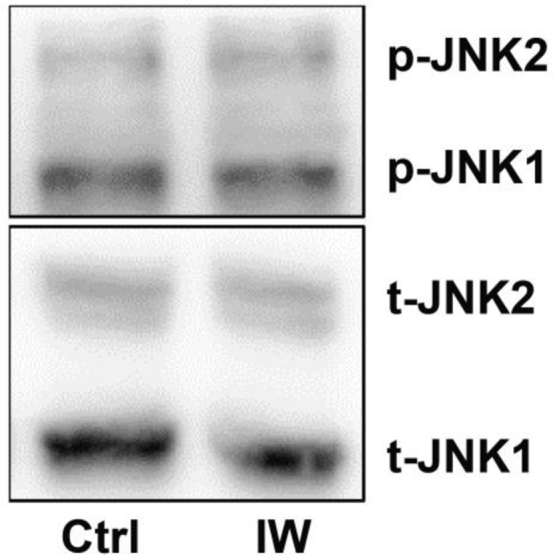
Representative western blot showing constitutive phosphorylation of JNK1/2 in ECs after 8 h. Along with untreated control the cells were exposed to 50 µmol/L of IW which did not change phosphorylation of JNK1/2.

The data reported include information on removal of endothelium from rat aorta (Fig. 1), endothelium-dependent relaxation with acetylcholine and norepinephrine induced wall tension of aorta (Fig. 2), dose–response curves for IW and captopril for inhibition of pro-MMP2 expression in ECs and SMCs (Fig. 3), inhibition of pro-MMP2 expression in rat aorta by IW and captopril (Fig. 4) and phosphorylation of JNK in presence of IW in ECs (Fig. 5).

## 2 Experimental design, materials and methods

### 2.1 Experiments on isolated rat aorta

#### 2.1.1 Histological staining

Hematoxylin–eosin staining was performed as previously described [2]. Briefly, after removal of paraffin with xylene (3×5 min), tissue sections were hydrated with series of alcohol (100% to 40%). After staining with hematoxylin (10 min) and eosin (2 min) sections were washed in demineralized water and dehydrated again with series of alcohol (40–100%). Sections were then cleared with xylene (3×5 min) and embedded with DePeX (Serva, Heidelberg, Germany).

#### 2.1.2 Mulvany myograph

Vessel tone was assessed *ex-vivo* as previously described [3]. Briefly, before experimentation, 4 mm of aortic section were prepared by removal of perivascular fat. After preconstriction of aorta with potassium–chloride (123.7 mmol/L KCl) and with noradrenaline (max. 10 µmol/L) endothelium-dependent relaxation was assessed in response to acetylcholine (10 µmol/L) and endothelium-independent relaxation with sodium nitroprusside (with 3.5 µmol/L). Relaxation of aorta was calculated as percent of maximum preconstriction with noradrenaline.

### 2.2 Cell culture and treatment

Commercially purchased ECs and SMCs from rat aorta were used for the study (Cell Applications Inc. California, USA). The cells were grown in T-75 culture flasks in supplemented medium and sub-cultured into 12-well culture plates for experimentation. The cells were treated either with ANGII or ANGI for 8 h with or without inhibitors. Cell treatments were performed in non-supplemented serum free medium.

### 2.3 Western blot

Western blot to quantitatively assess latent and active forms of MMP2 was performed as previously described [4]. Briefly, 10 µg of total protein was loaded on 10% SDS-PAGE gel. After electrophoretic separation, semi-dry transfer system was used to transfer protein to a nitrocellulose membrane. The membranes were blocked with 5% milk in TBS-Tween and stained using primary anti-MMP2 antibody (Millipore MAB3308) overnight at 4 °C with a gentle agitation. After staining with secondary antibodies, fluorescence was detected with PeqLAB imager and bands were quantified using Image J software.
